# The Blind as Seen through Blind Eyes

**Published:** 1897-09

**Authors:** 


THE BLIND AS SEEN THROUGH BLIND EYES. By MAURICE DE LA SIZERANNE. Authorised translation from the second French edition, by F. Park Lewis, M. D., Member of the Board of Trustees of the New York Institution for the Blind. Duodecimo, pp. 170. New York: G. P. Putnam's Sons, 27 West Twenty-third street. 1893.

This is an interesting work, dealing with the characteristics of the blind, physically, intellectually and morally, with Valentine Haüy and his work, with the school for the blind and with the blind in society, as viewed by a Frenchman who himself was blind. The story is told in a most fascinating way and every friend of the blind should read it. A. A. H.

